# A simple molecular technique for distinguishing species reveals frequent misidentification of Hawaiian corals in the genus *Pocillopora*

**DOI:** 10.7717/peerj.4355

**Published:** 2018-02-08

**Authors:** Erika C. Johnston, Zac H. Forsman, Robert J. Toonen

**Affiliations:** Hawai‘i Institute of Marine Biology, University of Hawai‘i at Mānoa, Kāne‘ohe, HI, United States of America

**Keywords:** Species distribution, Scleractinia, Phenotypic polymorphism, Kāne’ohe Bay, Northwest Hawaiian Islands

## Abstract

Species within the scleractinian genus *Pocillopora* Lamarck 1816 exhibit extreme phenotypic plasticity, making identification based on morphology difficult. However, the mitochondrial open reading frame (mtORF) marker provides a useful genetic tool for identification of most species in this genus, with a notable exception of *P. eydouxi* and *P. meandrina*. Based on recent genomic work, we present a quick and simple, gel-based restriction fragment length polymorphism (RFLP) method for the identification of all six *Pocillopora* species occurring in Hawai‘i by amplifying either the mtORF region, a newly discovered histone region, or both, and then using the restriction enzymes targeting diagnostic sequences we unambiguously identify each species*.* Using this approach, we documented frequent misidentification of *Pocillopora* species based on colony morphology. We found that *P. acuta* colonies are frequently mistakenly identified as *P. damicornis* in Kāne‘ohe Bay, O‘ahu. We also found that *P. meandrina* likely has a northern range limit in the Northwest Hawaiian Islands, above which *P. ligulata* was regularly mistaken for *P. meandrina*.

## Introduction

Species in the scleractinian genus *Pocillopora* Lamarck 1816 are known to exhibit extreme phenotypic plasticity (Pinzón et al., 2013; Marti-Puig et al., 2014; Paz-García et al., 2015a; Paz-García et al., 2015b; Gélin et al., 2017), making identification in the field difficult, particularly when colonies are small. For example, individuals displaying the classic *P. meandrina* Dana 1846 morphology in the Society Islands were recently targeted for a population genetic study; however, genetic sequencing of the widely used mitochondrial open reading frame (mtORF) marker (Flot et al., 2008) revealed the presence of six different genetic lineages (Edmunds et al., 2016).

While the mtORF marker (Flot et al., 2008) has been used to delineate up to 16 putative species of *Pocillopora* (Gélin et al., 2017), this marker does not delineate all species. For example, *P. meandrina* and *P. eydouxi* Milne Edwards 1860 share an identical sequence at this locus (mtORF type 1 (Pinzón et al., 2013)) even though genomic data have recently revealed that these lineages are distinct, and valid species (Johnston et al., 2017). Additionally, Schmidt-Roach et al. (2014a) found that *P. eydouxi* colonies have a styloid columella in Eastern Australia, whereas *P. meandrina* colonies present mostly convex, oval columellas.

Based on recent genomic work (Johnston et al., 2017), we hypothesized that we could develop a molecular test to delineate Hawaiian *Pocillopora* species. Here, we present a fast, simple, and inexpensive assay based on restriction fragment length polymorphism (RFLP) of PCR amplicons that unambiguously differentiates all six *Pocillopora* species found in Hawai‘i. We hypothesized that we could use this molecular assay to show that many species are currently misidentified with traditional gross colony morphology-based approaches using two examples. First, we examined the distribution of *P. meandrina* and *P. ligulata* Dana 1846 across the Hawaiian Archipelago, as we hypothesized that the latter, less commonly documented species might often be mistaken as the former. Second, we described the distribution of *P. acuta* Lamarck 1816 and *P. damicornis* (Linnaeus 1758) in Kāne‘ohe Bay, O‘ahu. Based on recent work by Schmidt-Roach et al. (2013), we hypothesized that the majority of the colonies previously referred to as *P. damicornis* in Kāne‘ohe Bay (e.g., Mayfield et al., 2010; Gorospe & Karl, 2013; Putnam & Gates, 2015) were actually *P. acuta.*

## Materials and Methods

### Restriction length polymorphism assays

To differentiate individuals of mtORF type 1 (i.e., *Pocillopora eydouxi* and *P. meandrina*; 978 bp) from all other species of *Pocillopora* (species names following Schmidt-Roach et al. (2014a) and mtORF types following Pinzón et al. (2013)), we aligned 50 mtORF sequences taken from Flot et al. (2008), Pinzón et al. (2013) and Johnston et al. (2017), spanning a wide geographic range, using *Geneious Alignment* in GENEIOUS 6.1.8. In this alignment, we identified a SNP (adenine; 676 bp) fixed for all individuals of mtORF type 1. We then amplified the mtORF region using the FatP6.1 (5′-TTTGGGSATTCGTTTAGCAG-3′) and RORF (5′-SCCAATATGTTAAACASCATGTCA-3′) primers of Flot et al. (2008), and digested the PCR product with the *Sac*I restriction enzyme (Table 1). PCR mixes contained 7.5 μL of BioMix (Bioline Ltd., London, UK), 0.195 μL of each forward and reverse primer (10 μM), 0.14 μL of BSA, 1.5 μL of template DNA (5–50 ng; extracted from coral biopsies using the Omega Bio-Tek EZ 96 Tissue DNA extraction kit; Omega Bio-Tek, Norcross, GA, USA), and 5.47 μL of deionized water to 15 μL final volume. Each PCR followed the cycling protocol of Flot et al. (2008), with a denaturation step of 60 s at 94 °C, followed by 40 cycles of 30 s at 94 °C, 30 s at 53 °C, and 75 s at 72 °C; thermocycling was followed by an incubation at 72 °C for 5 min. PCR products were then digested with 0.5 μL of *Sac*I restriction enzyme (New England BioLabs, Ipswich, MA, USA) and 0.5 μL of the 10X NEBuffer 1.1 for 1 hr at 37 °C, followed by heat inactivation at 65 °C for 20 min (Table 1). Three microliters of the digested products were run on a 2% TAE-agarose gel for 1.5 hr at 70 V.

**Table 1:** Summary of amplicon, restriction enzyme, restriction site, and respective fragment sizes (bp, base pairs) differentiating Hawaiian *Pocillopora* species.

Species	Amplicon	Restriction enzyme site	Fragment sizes (bp)	Amplicon	Restriction enzyme site	Fragment sizes (bp)	Cuts other species? Y/N (Fragment sizes bp)
*P. eydouxi*	(1) mtORF	*Sac*I GAGCT’C C’TCGAG	298, 680	(2) PocHistone	*Xho*I C’TCGAG GAGCT’C	287, 382	N
*P. meandrina*	(1) mtORF	*Sac*I GAGCT’C C’TCGAG	298, 680	(2) PocHistone	*Xho*I C’TCGAG GAGCT’C	669	N
*P. verrucosa*	(1) mtORF	*Aci*I C’CGC GGC’G	209, 338, 431				Y, all species (430, 548)
*P. ligulata*	(1) mtORF	*Alw*NI CAGNNN’CTG GTC’NNNCAG	467, 511				Y, mtORF 11 (467, 511)
*P. acuta*	(1) mtORF	*Nla*IV GGN’NCC CCN’NGG	30, 171, 315, 462	(2) mtORF	*Tsp*45I ‘GTSAC CASTG’	978	Y, *Nla*IV: mtORF 1, 2, 6, 8 (201, 315, 462) Y, *Nla*IV: mtORF 3^i^ (194, 784) Y, *Nla*IV: mtORF 3 (516, 462)
*P. damicornis*	(1) mtORF	*Nla*IV GGN’NCC CCN’NGG	30, 171, 315, 462	(2) mtORF	*Tsp*45I ‘GTSAC CASTG’	530, 448	Y, *Nla*IV: mtORF 1, 2, 6, 8 (201, 315, 462) Y, *Nla*IV: mtORF 3^i^ (194, 784) Y, *Nla*IV: mtORF 3 (516, 462) Y, *Tsp*451: mtORF 3^f^ (204, 774)

To then differentiate *P. eydouxi* from *P. meandrina*, we aligned 27 total sequences of *P. damicornis, P. acuta, P. verrucosa, P. meandrina, P. eydouxi, Pocillopora* sp. B, *P. ligulata*, and mtORF type 11 using *Geneious Alignment* in GENEIOUS 6.1.8 for the histone 3 region (669 bp) discovered in Johnston et al. (2017). The first half of the gene was identified as an open reading frame of unknown function and contains the SNP (thymine; 279 bp) fixed for *P. eydouxi*, which falls in the second position of the amino acid leucine; all other *Pocillopora* examined in this study for this region have guanine at this position, resulting in the amino acid arginine. The latter half of the region (337–659 bp) mapped to partial histone 3 genes from cnidarians *Plesiastrea versipora* (accession number: HQ203519; Huang et al., 2011) and *Nematostella vectensis* (accession number: XM_001633243; Putnam et al., 2007). DNA from individuals of mtORF type 1 (*P. meandrina* and *P. eydouxi*) were amplified using novel primers for this histone 3 region (PocHistoneF: 5′-ATTCAGTCTCACTCACTCACTCAC-3′ and PocHistoneR: 5′-TATCTTCGAACAGACCCACCAAAT-3′; accession numbers: MG587096–MG587097) (Table 1). PCR mixes were prepared as described above, and the following thermocycling protocol was used: denaturation for 60 s at 94 °C, followed by 40 cycles of 30 s at 94 °C, 30 s at 53 °C, and 60 s at 72 °C, with a final elongation step of 5 min at 72 °C. PCR products were digested with 0.5 μL of *Xho*I restriction enzyme (New England BioLabs, Ipswich, MA, USA) and 0.5 μL of 1X CutSmart^®^ buffer for 1 hr at 37 °C, followed by heat inactivation at 65 °C for 20 min (Table 1). Three microliters of the digested products were run on a 2% TAE-agarose gel for 1.5 h at 70 V.

*Pocillopora verrucosa* (Ellis and Solander 1786) (mtORF types 3a, 3b, 3c, 3d, 3e, 3f, 3g, 3h, 3i, 3j; Pinzón et al., 2013) was differentiated from all other *Pocillopora* species using the mtORF alignment described above, in which *P. verrucosa* was found to have two fixed SNPs (cytosine and guanine; 210–11 bp), and all *Pocillopora* species share this same restriction site at 547 bp. The mtORF region (Flot et al., 2008) was amplified and digested using 0.5 μL of the *Aci*I restriction enzyme (New England BioLabs, Ipswich, MA, USA) and 0.5 μL of 1X CutSmart^®^ buffer for 1 hr at 37 °C, followed by heat inactivation at 65 °C for 20 min (Table 1). Three microliters of the digested products were run on a 2% TAE gel for 1.5 h at 70 V.

*Pocillopora ligulata* was differentiated from all other *Pocillopora* species using the mtORF alignment described above. In the mtORF alignment, *P. ligulata* and mtORF type 11 (a haplotype which thus far has only been found in the Society Islands (Forsman et al., 2013; Gélin et al., 2017)) were both found to have a fixed SNP (cytosine; 462 bp). The mtORF region (Flot et al., 2008) was amplified and digested using 0.5 μL of the *AlwN*I restriction enzyme (New England BioLabs, Ipswich, MA, USA) and 0.5 μL of 1X CutSmart^®^ buffer for 1 hr at 37 °C, followed by heat inactivation at 80 °C for 20 min (Table 1). Three microliters of the digested products were run on a 2% TAE-agarose gel for 1.5 hrs at 70 V.

*Pocillopora damicornis* and *P. acuta* (mtORF type 4 and 5, respectively) were differentiated from all other species of *Pocillopora* using the mtORF alignment described above, in which both species were found to share a restriction site (GGN’NCC; 314, 344, and 515 bp) at three locations, while *P. meandrina, P. eydouxi, P. ligulata,* and mtORF type 8 share this same restriction site at two locations (314 and 515 bp). The mtORF region (Flot et al., 2008) was amplified and the PCR product digested with 0.5 μL of the *Nla*IV restriction enzyme (New England BioLabs, Ipswich, MA, USA) and 0.5 μL of 1X CutSmart^®^ buffer for 1 hr at 37 °C, followed by heat inactivation at 65 °C for 20 min (Table 1). Three microliters of the digested products were run on a 2% TAE-agarose gel for 1.5 hrs at 70 V.

In the mtORF alignment described above, *P. damicornis* was found to have a fixed SNP (cytosine; 534 bp), while all other *Pocillopora* have adenine in this position. Thomas et al. (2016) used this same SNP to differentiate *P. damicornis* from all other *Pocillopora* in Western Australia in their fluorescence-based quantitative real-time PCR (qPCR) assay. To differentiate *P. damicornis* from *P. acuta*, DNA from *P. damicornis* and *P. acuta* were amplified again with the primers of Flot et al. (2008) and digested with 0.5 μL of the *Tsp*45I restriction enzyme (New England BioLabs, Ipswich, MA, USA) and 0.5 μL of 1X CutSmart^®^ buffer for 1 hr at 65 °C (Table 1). Three microliters of the digested products were run on a 2% TAE-agarose gel for 1.5 hrs at 70 V.

### *Pocillopora* distribution patterns in Hawai‘i

*Pocillopora* samples (<10 cm) were collected from colonies displaying *P. meandrina* morphology (with a subset of colonies verified by J Maragos or P Jokiel) across the Hawaiian Islands haphazardly at depths of 2–20 m on either NOAA research cruises or on SCUBA from shore dives between 2005 and 2016 (Hawai‘i Island, 104; Mau‘i, 59; Lana‘i, 15; Moloka‘i, 10; O‘ahu, 189; Kaua‘i, 16; Ni‘ihau, 26; Nihoa, 32; Kānemiloha‘i (French Frigate Shoals), 44; Pūhāhonu (Gardner Pinnacles), 28; Nalukākala (Maro Reef), 10; Kauō (Laysan Island), 35; Holoikauaua (Pearl and Hermes Atoll), 47; Pihemanu (Midway Atoll), 52; Moku Pāpapa (Kure Atoll), 24). All tissue samples were stored in either salt-saturated DMSO (dimethyl sulfoxide) buffer (Gaither et al., 2011) or >95% ethanol until DNA was extracted. Genomic DNA was extracted from tissues using the Omega E-Z 96 Tissue DNA Kit (Omega Bio-Tek, Norcross, GA, USA). PCRs were prepared as described above and PCR products were first digested with the *Sac*I restriction enzyme. Individuals of mtORF type 1, i.e., either *P. eydouxi* or *P. meandrina*, were then amplified using the PocHistone marker and digested with the *Xho*I restriction enzyme to differentiate *P. eydouxi* from *P. meandrina*. Those samples that were neither *P. meandrina* nor *P. eydouxi* were then digested with the *AlwN*I and restriction enzyme to determine if they were *P. ligulata.* And finally, samples that were not identified as *P. meandrina*, *P. eydouxi*, or *P. ligulata* were digested with the *Aci*I restriction enzyme to determine if they were *P. verrucosa*.

Twenty-five colonies, varying in length from 5–30 cm, and displaying the full range of *P. acuta/P. damicornis* morphology (Schmidt-Roach et al., 2014a), were sampled from a 100 m^2^ area in July 2017 from Kāne‘ohe Bay, O‘ahu (21.456449, −157.794413), at a depth of 4 m, under HIMB special activity permit 2018-3, to determine the relative abundance of these species in the Bay. All tissue samples were stored in salt-saturated DMSO, DNA was extracted as described above, PCRs were prepared as described above, and PCR products were first digested with the *Nla*IV restriction enzyme to ensure that all samples were either *P. acuta* or *P. damicornis*. New PCRs were carried out and the amplicons were digested with the *Tsp*45I restriction enzyme to differentiate between the two species.

## Results

**Figure 1:**
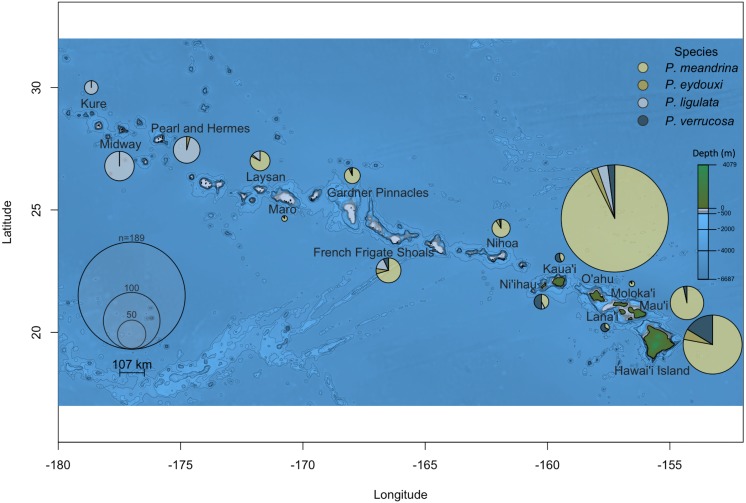
*Pocillopora* species composition across the Hawaiian Islands for samples collected from colonies demonstrating *P. meandrina* morphology. The size of the pie chart is proportional to the number of individuals sampled per island. *Pocillopora* species are represented by different colors, specifically: *P. meandrina*, light yellow; *P. eydouxi*, dark yellow; *P. ligulata*, light blue; and *P. verrucosa*, dark blue.

Of the 691 samples displaying classical *P. meandrina* morphology collected across the Hawaiian Islands, one-third (222 samples) were not *P. meandrina*. Two striking and previously unknown patterns of *Pocillopora* distribution stood out. The first was in the Main Hawaiian Islands. Despite the low number of samples collected from Lana‘i, Kaua‘i, and Ni‘ihau, more were *P. verrucosa* than any other *Pocillopora* species (10/15 on Lana‘i, 9/16 on Kaua‘i, and 13/26 on Ni‘ihau; Fig. 1). The second surprising pattern was discovered in the Northwest Hawaiian Islands, where none of the 123 presumed *P. meandrina* samples collected from the three most northerly islands (Pearl and Hermes, Midway, and Kure) were correctly identified; nearly all were *P. ligulata*, though two collected from Pearl and Hermes were *P. eydouxi* (Fig. 1).

Previously, all fine branched *Pocillopora* species in Hawai‘i were identified as *P. damicornis*, but the presence of *P. acuta* was also recently confirmed (Schmidt-Roach et al., 2014b; Johnston et al., 2017). This discovery prompted us to look at whether both species were present in Hawai‘i, and if so, what the relative frequency of each in Kāne‘ohe Bay was, the most studied location in the Hawaiian Islands. Of the 25 samples collected from a 100 m^2^ area in Kāne‘ohe Bay, O‘ahu, 24 were *P. acuta* and one was *P. damicornis*, indicating that both species are present, but at very different relative abundances, at least from the wave-exposed, barrier reef site from which these samples were collected.

## Discussion

**Figure 2:**
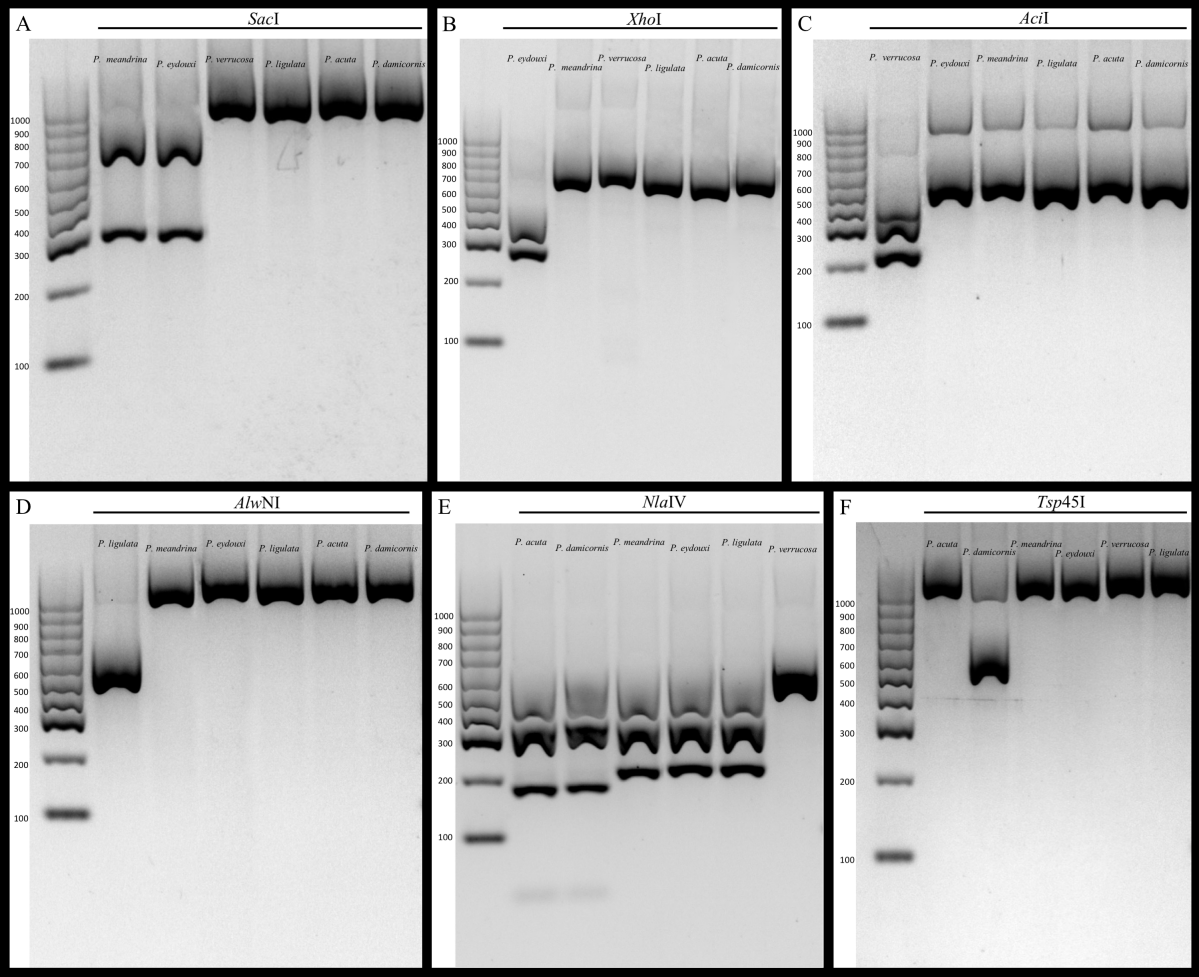
Gel images of amplicons digested with restriction enzymes. (A) mtORF amplification and digestion with *Sac*I restriction enzyme differentiates mtORF type 1 (*P. meandrina* and *P. eydouxi*) from all other *Pocillopora*. (B) PocHistone amplification and digestion with *Xho*I restriction enzyme differentiates *P. eydouxi* from all other *Pocillopora*. (C) mtORF amplification and digestion with the *Aci*I restriction enzyme differentiates *P. verrucosa* from all other *Pocillopora*. (D) mtORF amplification and digestion with the *AlwN*I restriction enzyme differentiates *P. ligulata* from all other *Pocillopora*. (E) mtORF amplification and digestion with the *Nla*IV restriction enzyme differentiates *P. acuta* and *P. damicornis* from all other *Pocillopora*. (F) mtORF amplification and digestion with the *Tsp*45I restriction enzyme differentiates *P. damicornis* from all other *Pocillopora*.

We demonstrate that our assay works to identify all the currently known species of *Pocillopora* in the Hawaiian Archipelago, and expect that it will be equally useful for other locations throughout the Pacific. However, additional testing by labs in other locations will be needed to confirm reliability among different locations, and some regional modifications are undoubtedly required. For example, the same restriction enzyme used for *P. ligulata* (*Alw*NI) in Hawai‘i can be used for haplotype 11 (Forsman et al., 2013) in French Polynesia because *P. ligulata* does not occur there, but shares the same identifying cut site as haplotype 11 from Moorea (Edmunds et al., 2016). Likewise, Thomas et al. (2016) developed a fluorescence-based quantitative real-time PCR assay that distinguishes *P. damicornis* from all other *Pocillopora* species in Western Australia. Our RFLP assay relies on the same SNP to distinguish *P. damicornis* from *P. acuta* indicating that replicability is likely inherent in our assay. In contrast, Torda et al. (2013) previously published a RFLP assay that distinguishes *P. damicornis* and *P. acuta* from all other *Pocillopora* in Australia using the putative control region. Despite a concerted effort, these primers failed to amplify our samples, however; this may be due to the fact that we did not purchase the proprietary Qiagen Multiplex PCR Kit used by Torda et al. (2013) in their protocol, or it may be due to regional differences in the sequences selected to differentiate the species that limit the utility of primers. Whatever the reason, our gel-based RFLP approach provides a cost-effective assay without proprietary reagents that quickly allows discrimination amongst *Pocillopora* species using simple PCR amplification followed by digestion with widely available restriction enzymes (Fig. 2). The sequencing of 691 samples at $3.50 in both directions for two genes would cost approximately $10,000, whereas the cost of all enzymes and reagents used herein was only $500; this represents a cost savings of nearly 95%.

**Figure 3:**
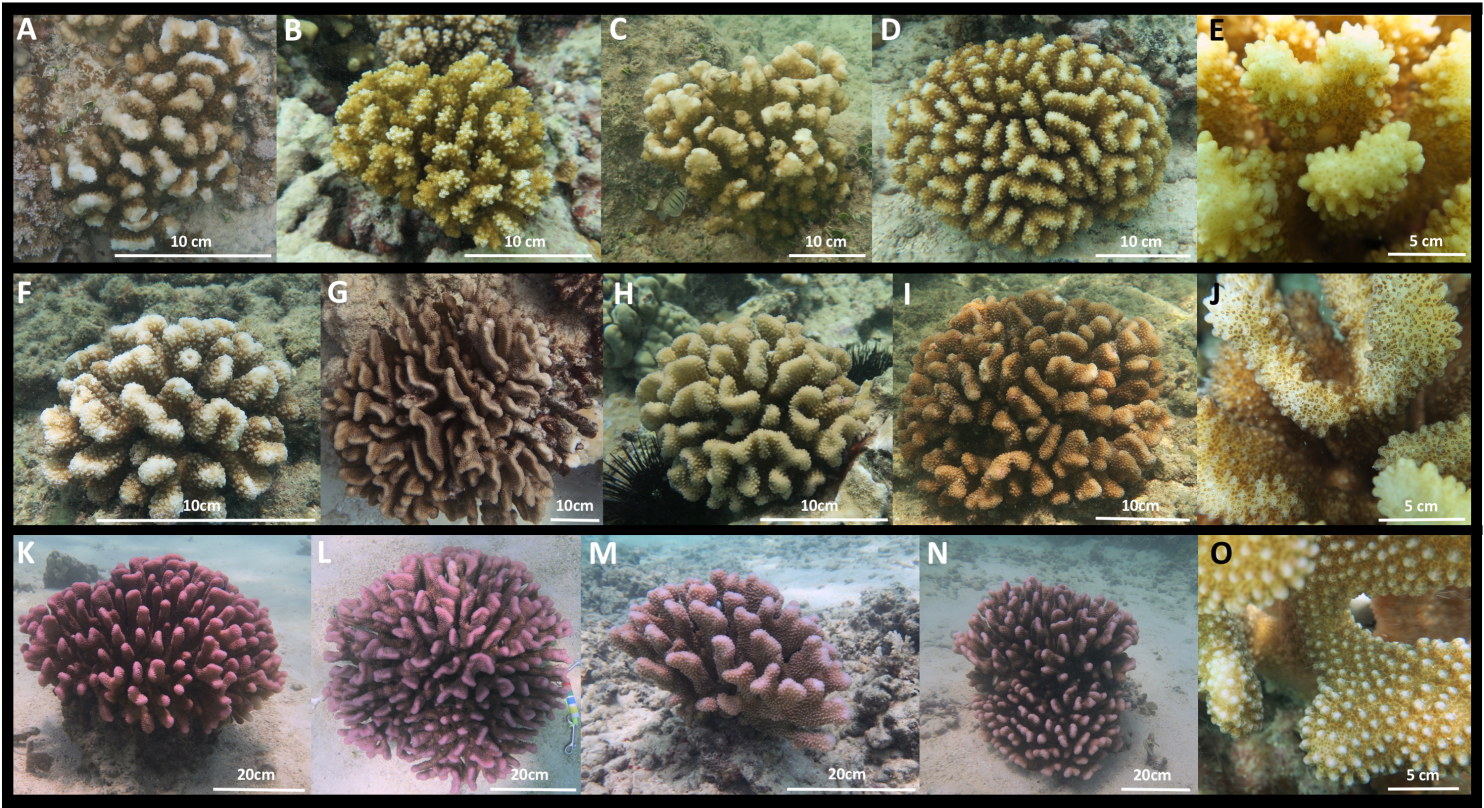
Images of *Pocillopora ligulata* colonies, (A)–(E); *P. meandrina* colonies, (F)–(J); and *P. eydouxi* colonies, (K)–(O) from O‘ahu, Hawai‘i.

The occurrence of *P. ligulata*, and complete absence of *P. meandrina,* from the three most northern Northwest Hawaiian Islands is striking. Very little is known about *P. ligulata*. This species is described as having branches with flattened ends and truncated tips that irregularly radiate; the verrucae are widely spaced and irregular (Veron & Stafford-Smith, 2000; Fig. 3). In contrast, *P. meandrina* has flattened branches that centrally radiate, with neat and uniform verrucae (Veron & Stafford-Smith, 2000; Fig. 3). Genetic surveys to date document *P. ligulata* only in the Hawaiian Islands (Flot et al., 2008; Forsman et al., 2013; Marti-Puig et al., 2014; Gélin et al., 2017), and its distribution, ecology, and reproductive biology is entirely unknown. *Pocillopora meandrina* is a widespread species with a distribution spanning from the Eastern Pacific to the east coast of Africa (Flot et al., 2008; Forsman et al., 2013; Schmidt-Roach et al., 2014b; Paz-García et al., 2015a; Gélin et al., 2017). It is hermaphroditic and its reproductive behavior has been characterized in Hawai‘i (Cox, Krupp & Jokiel, 1998) and Australia (Schmidt-Roach et al., 2012), though little is known about its ecology. The Northwest Hawaiian Islands are subtropical and contain the northernmost coral atolls in the world. Colonies displaying the typical *P. meandrina* morphology from the three most northern atolls (Pearl and Hermes, Midway, and Kure) turned out not to include any individuals of this species. Instead, collections from the three northernmost atolls were dominated by *P. ligulata*. Although little is known about the ecology of either *P. ligulata* or *P. meandrina*, based on these data we hypothesize that *P. meandrina* may have a northern range limit to the south of Pearl and Hermes, while *P. ligulata* may be better adapted to the northern edges of the subtropics. Future studies that sample corals across a range of habitat types (e.g., depths, reef types, temperatures, etc.) may reveal that these species are significantly associated with different habitat types (as shown by Mayfield et al., 2015; Mayfield, Chen & Dempsey, 2017a; Mayfield, Chen & Dempsey, 2017b), but we lacked the environmental data from which individual colonies were sampled to perform an equivalent analysis here.

The extreme phenotypic plasticity exhibited by *P. damicornis* and *P. acuta* has obscured the understanding of the distribution, ecology, and reproductive biology of these two species until very recently (Schmidt-Roach et al., 2013; Schmidt-Roach et al., 2014b). They are often found in the same environments (albeit with differing abundance by depth and exposure (Mayfield et al., 2015; Mayfield, Chen & Dempsey, 2017a; Mayfield, Chen & Dempsey, 2017b)), typically more sheltered locations such as lagoons and back reefs, but can also be found in more exposed environments (Schmidt-Roach et al., 2013; Schmidt-Roach et al., 2014a). These species generally share a sympatric distribution from Hawai‘i to the Indian Ocean, however *P. acuta* extends its range into the Arabian Gulf (Pinzón et al., 2013). Most studies to date have unknowingly lumped the two species, but both *P. damicornis* and *P. acuta* are hermaphroditic and colonies have been documented to both brood and spawn (Richmond & Jokiel, 1984; Jokiel, Ito & Liu, 1985; Ward, 1995; Permatav & Kinzie, 2000; Fan et al., 2002; Combosch & Vollmer, 2013; Massé et al., 2013; Schmidt-Roach et al., 2014b).

**Figure 4:**
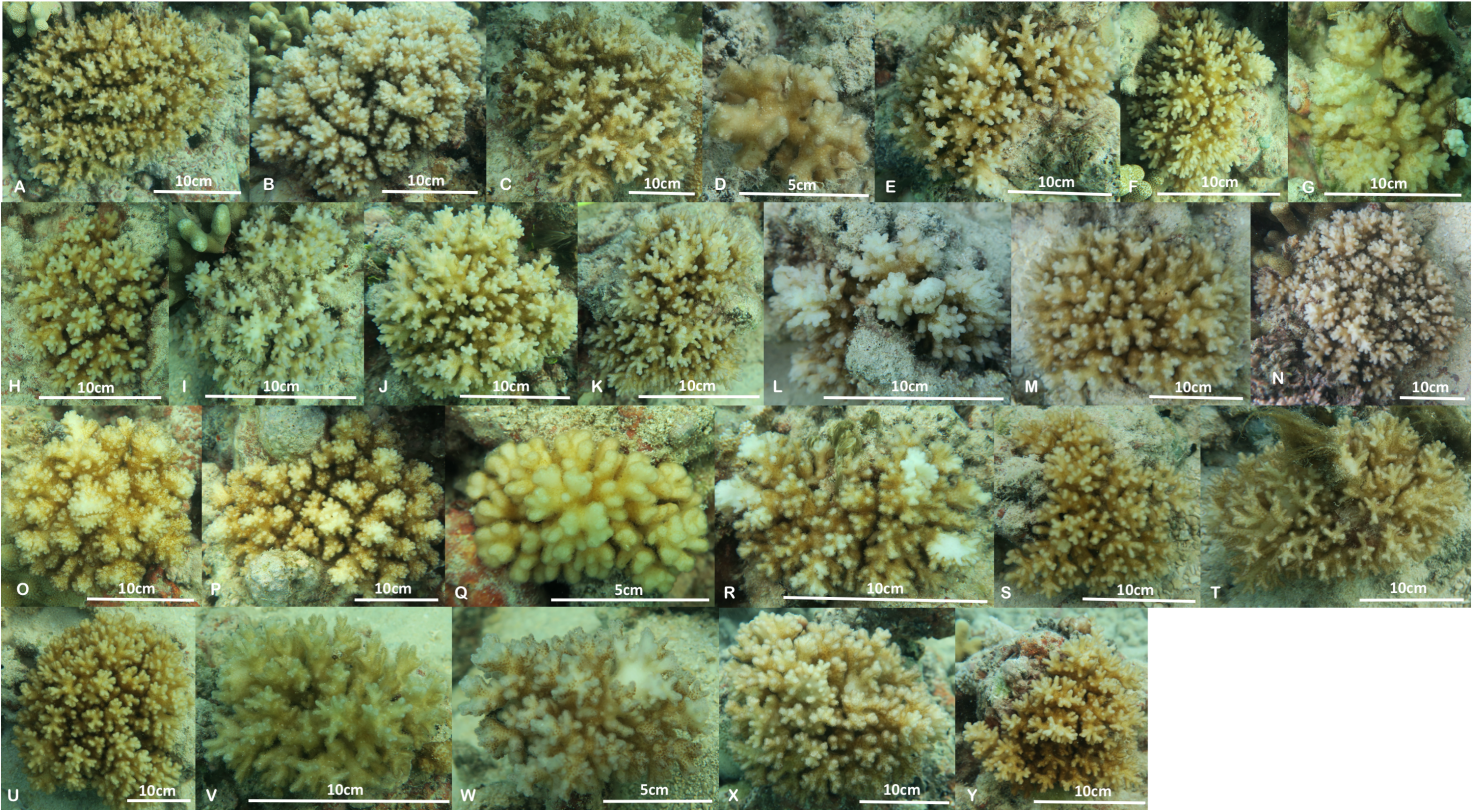
Images of the 25 colonies of *Pocillopora acuta* and *P. damicornis* collected from Kāne‘ohe Bay, O‘ahu. Colony L is *P. damicornis*, all other colonies are *P. acuta*.

Although only *P. damicornis* was previously known from Hawai‘i, there are two well-characterized ecomorphs, types Y and B, previously documented from Kāne‘ohe Bay. Type B is darker brown, with fine branch tips that are white in color, and releases small planulae on the full moon, whereas Y is stouter, more yellow in color with even pigmentation, and produces larger planulae released on the new moon (Jokiel, Ito & Liu, 1985). Both types were historically common and co-occurred on the reef flats of Kāne‘ohe Bay (Richmond & Jokiel, 1984), although there is nearly continuous variation from one extreme to the other, making identification of intermediate morphologies extremely difficult (PL Jokiel & RH Richmond, pers. comm., 2013). Further, type Y was essentially wiped out during the freshwater kill event in 1988 (Jokiel et al., 1993; Bahr, Jokiel & Toonen, 2015) and has not recovered in the bay since that time (P Jokiel, pers. comm., 2013). Based on the descriptions of the Y and B types, it is not clear if they correspond to *P. damicornis* and *P. acuta*, or rather stout vs fine branch morphology that both species appear able to exhibit. Regardless, the frequent misidentification of these species is almost certain to have obscured differences in habitat preference and reproduction likely to exist between them (e.g., Mayfield et al., 2015; Mayfield, Chen & Dempsey, 2017a; Mayfield, Chen & Dempsey, 2017b). The assay developed here will be useful for answering fundamental questions regarding reproductive isolation and habitat differentiation of these two species that are very recently diverged (less than a million years) and frequently lumped in previous studies (Thomas et al., 2014; Johnston et al., 2017). Insofar as our site is representative of wave dominated, barrier reef ecosystems in Kāne‘ohe Bay, our findings indicate that *P. acuta* is currently far more prevalent than *P. damicornis* (Fig. 4). Now that it is possible to positively identify the closely related species in this genus using our genetic assay, it will be interesting to determine their habitat preferences and distribution in Hawai‘i, and whether the relative abundance of the species changes over time or space.

## Conclusions

Here, we present an assay that allows rapid and unambiguous identification of all six species of *Pocillopora* present in Hawai‘i, which we hope will work anywhere these species are found. We present two cases where samples identified morphologically were misidentified to highlight the utility of this approach. Taxonomic confusion can impact a wide range of studies and the ability to rapidly and cost-effectively distinguish among species of *Pocillopora* will benefit future studies of population structure, ecology, biodiversity, evolution and conservation in this challenging genus.

##  Supplemental Information

10.7717/peerj.4355/supp-1 Data S1 Histone FASTA alignment raw data

10.7717/peerj.4355/supp-2 Data S2 mtORF FASTA alignment raw data mtORF haplotype number after Pinzón et al. (2013) indicated by either PZ# or Z#.

## Additional Information and Declarations

### Field Study Permissions

The following information was supplied relating to field study approvals (i.e., approving body and any reference numbers): Collection of coral tissue samples was approved by the State of Hawai’i, Department of Land and Natural Resources, Division of Aquatic Resources Special Activity Permit 2018-3.

### DNA Deposition

The following information was supplied regarding the deposition of DNA sequences: The accession numbers for the histone 3 region of *P. eydouxi* and *P. meandrina* are MG587096–MG587097. Sequence data can also be found in the Supplemental Information.

### Data Availability

The following information was supplied regarding data availability: The raw data and code are provided in Supplemental Files.
